# Use of Sodium-Glucose Co-Transporter-2 Inhibitors in Type 1 Diabetics: Are the Benefits Worth the Risks?

**DOI:** 10.7759/cureus.10076

**Published:** 2020-08-27

**Authors:** Tehreem Fatima, Surik Sedrakyan, Muhammad R Awan, Mst. Khaleda Khatun, Dibyata Rana, Nusrat Jahan

**Affiliations:** 1 Internal Medicine, California Institute of Behavioral Neurosciences & Psychology, Fairfield, USA

**Keywords:** diabetes mellitus type 1, sodium-glucose co-transporter 2 inhibitor, diabetes type 1

## Abstract

The mainstay of treatment for type 1 diabetes is insulin. The use of insulin for tight glycemic control is the key to preventing micro- and macrovascular complications, but it can also lead to hypoglycemic episodes. Therefore, there is a need for the introduction of a drug that can maintain glucose levels within a safe range without increasing the risk of hypoglycemia. For this reason, SGLT2 (sodium-glucose co-transporter-2) inhibitors has been a hot topic in the last couple of years. They have been proved very efficient in treating type 2 diabetes. Many trials on the safety and efficacy of SGLT2 inhibitors have been done on type 1 diabetics. Some other studies have also been done that prove their benefits in increasing arterial efficacy and reducing GFR (glomerular filtration rate). This review article discusses the benefits and risks. The literature search was performed using PubMed, and after applying the inclusion and exclusion criteria, 16 published papers were found. All relevant articles on the topic have been included. Our review has shown that the benefits of SGLT2 inhibitors outweigh their risks. Their benefits include good glycemic control, HBA1c (glycated haemoglobin) reduction, weight loss, and blood pressure improvement. Furthermore, improvement in GFR and arterial efficacy is also significant. Side effects such as UTI (urinary tract infection) and genital infection have been observed, but their incidence is low. However, DKA (diabetic ketoacidosis) and hypoglycemia are severe side effects that should be highlighted. Hypoglycemia can be prevented by strictly monitoring blood sugar levels. The patient must be educated and counseled about DKA and its symptoms. This will ensure the safety of the patient as euglycemic DKA can prove fatal if not diagnosed earlier. So, SGLT2 inhibitors can be used as an effective drug to control blood sugar levels in type 1 diabetes, especially in patients with a BMI higher than 30. It will not only achieve the treatment goals but can also decrease the morbidity and mortality of the patients. However, more studies need to be done to fully understand DKA caused by SGLT2 inhibitors.

## Introduction and background

According to 2019 statistics, type 1 diabetes mellitus accounts for five to ten percent of cases of diabetes in the United States, Canada, and Europe [1]. Worldwide, around 1.1 million children and adolescents suffer from type 1 diabetes. Type 1 diabetes is primarily caused by a lack of insulin production while type 2 diabetes mellitus (T2DM) is characterized by the resistance of body tissues to insulin. About 75% of people do not achieve the required glycemic control of HBA1c (glycated hemoglobin) = 7%, as recommended by the American Diabetes Association [2]. The mainstay of treatment of type 1 diabetes is lifestyle modification, which includes a change in diet and increased physical activity along with insulin replacement [3]. Achieving the required glycemic control is vital because failing to do so might lead to multiple complications such as micro- and macrovascular changes. On the other hand, tight glycemic control may give rise to hypoglycemic episodes, which result in cardiovascular complications in the long run [4]. Hence, a drug that can both control the blood sugar levels and eliminate the risk of hypoglycemia is crucial in the management of type 1 diabetes.

Several drugs have been studied over the past decade that can help to achieve this goal. One such drug is SGLT2 (sodium-glucose co-transporter-2) inhibitors, previously used effectively for type 2 diabetes and known to have a cardio-protective role in T2DM [5]. SGLT2 inhibitor acts by antagonizing SGLT2 receptors in the proximal tubule of the kidney, which is involved in the reabsorption of filtered glucose. This results in promoting renal excretion of glucose, thereby lowering elevated glucose levels [6]. This mechanism helps regulate the patient's blood sugar levels while simultaneously preventing hypoglycemia. Moreover, they can improve HBA1c and body weight among these patients [7]. However, there are several adverse effects, such as euglycemic DKA (diabetic ketoacidosis), UTI (urinary tract infection), genital infections, and AKI (acute kidney injury) [8]. The efficacy and safety of SGLT2 inhibitors in type 1 diabetics have been studied previously, but a comparison of all the risks and benefits of SGLT2 inhibitors and critical analysis on these have not been done.

This literature review aims to discuss the advantages and disadvantages of SGLT2 inhibitors in type 1 diabetes and to review whether benefits outweigh the risks. The most important aspect of this research is to analyze how beneficial it is to improve HBA1c at the expense of euglycemic DKA. We have critically analyzed this group of medications as these can be a groundbreaking addition to the typical regimen of insulin-only treatment for type 1 diabetes patients, especially for those with uncontrolled diabetes. 

## Review

Method

Literature was searched on PubMed. MeSH keywords used were "Diabetes Mellitus, Type 1" AND "Sodium-Glucose Transporter 2 Inhibitors". Studies were selected after applying the following inclusion/exclusion criteria.

The inclusion criteria were: 1) human subjects; 2) paper published in the English language; 3) study types that are clinical trials, observational studies, cohort studies, case-control studies, case reports, and systematic review; and 4) only meta-analysis and systematic review with full articles to be included.

The exclusion criteria were: 1) animal studies and 2) non-English literature.

Results

After applying the MeSH keywords "Diabetes Mellitus, Type 1" and "Sodium-Glucose Transporter 2 Inhibitors" a total of 97 articles were obtained. Of these articles, about 50 were removed as they were not specifying the disease and drug of interest. On secondary screening, further 31 articles were removed which consisted of book chapters, letters to the editor, review articles, and duplicates. The remaining 16 articles were used for our review article. The total number of participants in our study were 5,256.

Discussion

In this current review, we analyzed data from 5,256 patients with type 1 diabetes who used SGLT2 inhibitors, to look more closely at the risks and benefits of this drug in type 1 diabetes. For this reason, we have tried not only to include clinical trials on type 1 diabetes but also trials illustrating benefits, e.g., glomerular hyperfiltration and arterial efficacy, and case reports highlighting side effects, e.g., DKA, Fanconi Syndrome. These are summarized in Table 1.

**Table 1:** Table illustrating the baseline characteristics of the trials used in the review article DKA: diabetic ketoacidosis

Study/ reference		Subjects (N)	Study design		Study duration		Intervention
Mudaliar et al., 2012 [9]		10	RCT		Each participant in five treatment periods separated by five to 35 days		Remogliflozin Etobonate 50mg, 150mg, 500mg, placebo, prandial insulin
Parkins et al., 2014 [10]		40	RCT		8 weeks		Empagliflozin 25mg
Henry et al., 2015 [11]		70	RCT		2 weeks		Dapagliflozin 2.5mg, 5mg, 10mg, placebo
Pleber et al., 2015 [12]		75	RCT		4 weeks		Empagliflozin 2.5mg, 10mg, 25mg, placebo
Tomez et al., 2015 [13]		12	Before-after study		24 weeks		Dapagliflozin 10mg
Henry et al., 2015 [14]		35	RCT		18 weeks		Canagliflozin 100mg, 300mg
Dandona et al., 2018 [15]		833	RCT		52 weeks		Dapagliflozin 5mg, 10mg
Kaku et al., 2019 [16]		40	RCT		2 weeks		Ipragliflozin 25mg, 50mg, 100mg
Musso et al., 2019 [17]		3238	Meta-analysis		NA		Sotagliflozin
Mathieu et al., 2020 [18]		813	RCT		52 weeks		Dapagliflozin 5mg, 10mg
Benefits							
Arterial function							
Lunder et al., 2018 [19]		40	RCT		12 weeks		Empagliflozin
Glomerular hyperfiltration							
Cherney et al. [20]		40	RCT				Empagliflozin 25mg
Risks							
DKA							
Peters et al., 2015 [21]	7		Case-series		NA	SGLT2 inhibitors	
Tahir et al., 2015 [22]	1		Case-report		NA	SGLT2 inhibitors	
Markey et al, 2018 [23]	1		Case-report		NA	SGLT2 inhibitors	
Fanconi syndrome							
Khan et al., 2019 [24]	1			Case-report	NA	Canagliflozin	

Randomized control trials: safety and efficacy of SGLT2 inhibitors in type 1 diabetics

Glycemic Benefits of SGLT2 Inhibitors

Researchers of randomized control trials (RCT), established some endpoints to assess the glycemic benefits of SGLT2 inhibitors. They were glucose efficacy parameters and HBA1c levels.

**Figure 1:**
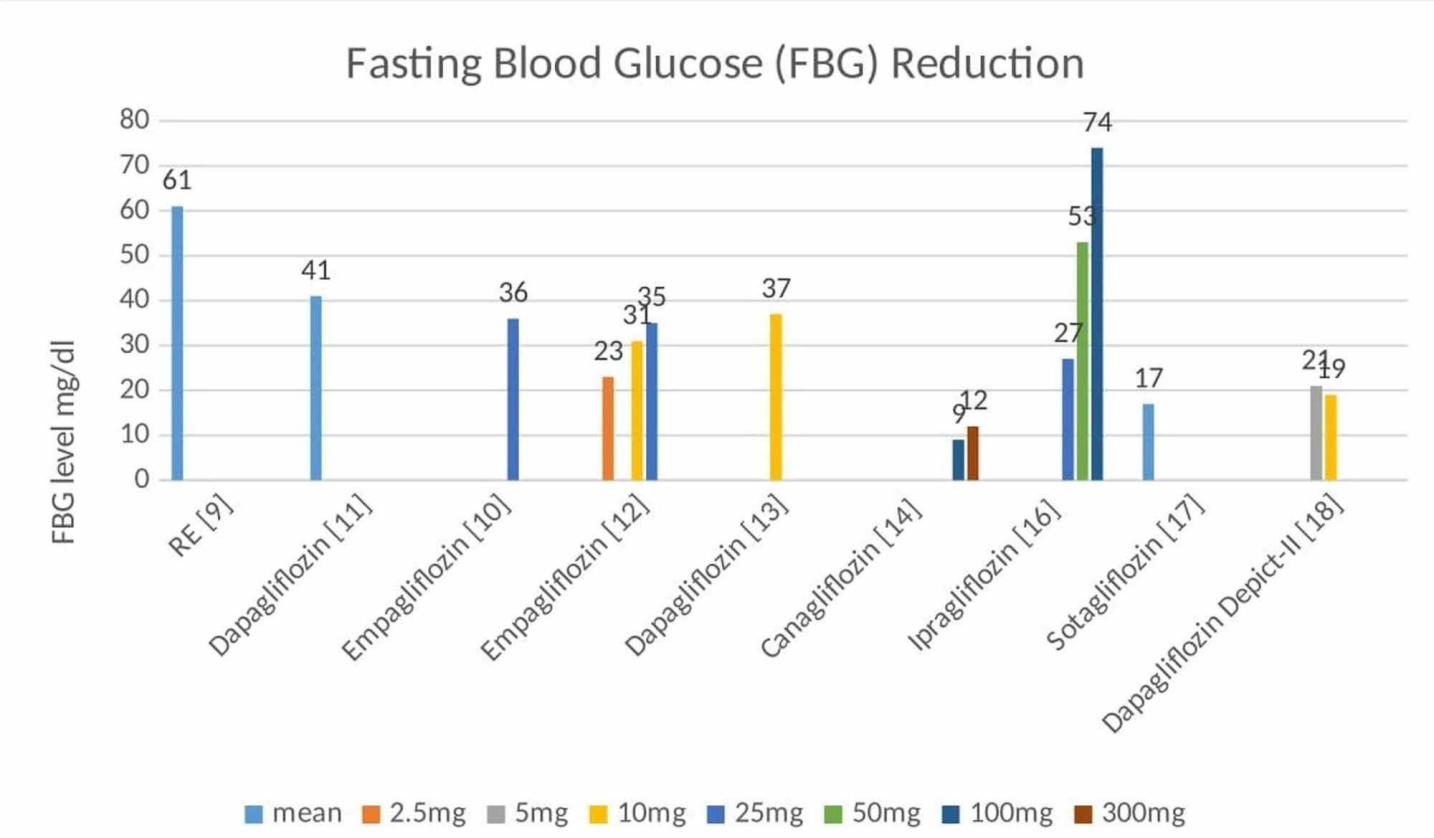
Fasting blood glucose level reduction as seen in all randomized control trials All values were adjusted according to placebo and baseline values. All values are FBG, except for RE [9], which is reduced incremental adjusted weighted mean glucose, and for dapagliflozin [11], which is 24-h daily average blood glucose.

Glucose efficacy: Almost all the trials reported glucose efficacy parameters by using fasting blood glucose (FBG) as mentioned in Figure 1. In all trials, the use of SGLT2 inhibitors was associated with a modest decrease in glucose levels. The most significant drop was observed in 100mg Ipragliflozin, where the adjusted mean difference to placebo was 74mg/dl, but this trial was done for two weeks [16].

**Figure 2:**
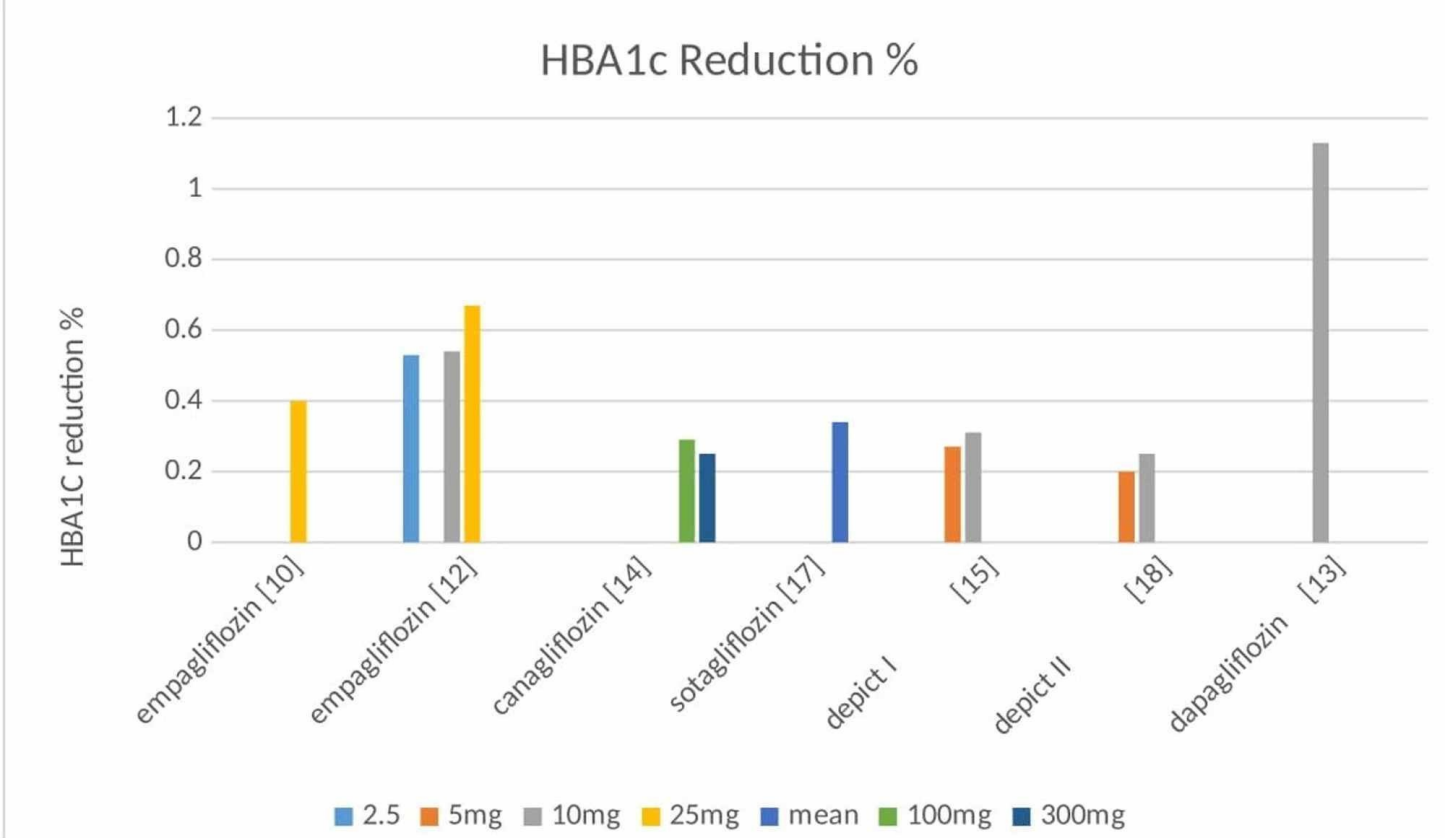
HBA1c reduction as seen in all randomized control trials

HBA1c levels: Similarly, most of the studies mentioned HBA1c. All studies showed a drop in their levels as seen in Figure 2. The most significant finding was seen with dapagliflozin 10mg, which showed a drop of about 1.13% as compared to placebo [13]. Some studies didn't mention HBA1c and used glucose control as the primary means of determining the endpoint. HBA1c is one of the most important factors in determining blood glucose control. Reduction in HBA1c for a longer period of time means fewer chances of microvascular and macrovascular complications [25].

Non-glycemic Benefits of SGLT2 Inhibitors

In addition to the beneficial effects related to improved glycemic control, SGLT2 inhibitors have several non-glycemic benefits, such as weight loss, insulin dose reduction, positive effect on blood pressure, increasing arterial efficacy, and a possible reno-protective effect.

Weight loss*:* Weight gain is a major problem for type 1 diabetics as insulin, which is the mainstay of treatment, causes weight gain. The effect of SGLT2 inhibitors on weight was explored in five studies. Weight reduction was seen in patients as shown in Table 2. The most significant finding was a decrease of up to 5% as compared to placebo [18]. Weight loss can be attributed to the loss of calories in the urine.

**Table 2:** Insulin use reduction and weight reduction with the use of SGLT2 inhibitors * Mean adjusted insulin dose reduction from baseline compared with placebo. Values are in %, IU, and mg as given in the original study. ** Bodyweight reduction is adjusted from baseline and placebo. Except for 417; which mentioned average weight decreased. Values are in % and kg.

Study/ reference	Mean adjusted insulin dose reduction from baseline*	Mean adjusted bodyweight reduction from baseline**
Parkins et al., 2014 [10]	9 units	2.6kg
Henry et al., 2015 [11]	−16.2%	NA
Pleber et al., 2015 [12]	−0.07 to −0.09 U/kg	1.5 to 1.6kg
Henry et al., 2015 [14]	100mg 9%	NA
	300mg 13%	
Dandona et al., 2018 (DEPICT-I) [15]	Reduction more for 10mg than 5mg	5mg 2.95%
		10mg 4.54%
Kaku et al., 2019 [16]	25mg 14%	
	50mg 18%	
	100mg 19%	
Musso et al., 2019 [17]	9%	3.54%
Mathieu et al., 2020 (DEPICT-II) [18]		5mg 4.42%
		10mg 5%

Insulin dose reduction: Insulin dose reduction was reported by seven studies in the dataset. All showed a significant reduction in insulin dose. Few studies reported mean change in insulin dose from, placebo while others mentioned mean change from the baseline. Nevertheless, the use of SGLT2 inhibitors was associated with a reduction in insulin dose as shown in Table 2.

Effects on blood pressure: Reduction in systolic blood pressure, which could be very beneficial for hypertensive patients. Only, two studies mentioned changes in blood pressure. A meta-analysis of sotagliflozin [17] mentioned a 4mm Hg drop, while the DEPICT-II trial observed an average 3mm Hg reduction with 5mg and 7mm Hg with 10mg dapagliflozin [18].

Side Effects

These trials not only assessed the benefits of SGLT2 inhibitors in type 1 diabetic patients but also looked closely at the safety profile. The adverse effects of SGLT2 inhibitors are summarized in Table 3.

**Table 3:** Summary of selected safety outcomes in clinical trials of SGLT2 inhibitors with type 1 diabetes. * Inability to perform daily activities ** Hypoglycemia value in % *** Rates of severe hypoglycemic episodes per 30 days

Dose /mg	Genital infections (%)	Severe hypoglycemia (number of events )*	Urinary tract infections (%)
Henry et al., 2015 [11} dapagliflozin			
1	7.7	0	0
2.5	0	0	6.7
5	7.1	0	0
10	0	0	0
Placebo	0	6.7%**	7.7
Pleber et al., 2015 [12] empagliflozin			
2.5	NA	0.9***	NA
10		1.1***	
25		1.2***	
Placebo		0.9***	
Henry et al., 2015 [14] canagliflozin			
100	9	8	5
300	2	3	4
Placebo	3	2	2
Dandona et al., 2018 [15] (DEPICT-I) dapagliflozin			
5	16	46	12
10	14	58	5
Placebo	3	85	8
Musso et al., 2019 [17]			
Sotagliflozin	3.12 times more risk	Lower risk of hypoglycemia than placebo ( 31%)	No relationship found
Mathieu et al., 2020 [18] (DEPICT-II) dapagliflozin			
5mg	11	97	9.2
10mg	10	84	5.2
Placebo	4	65	18

Most of the studies mentioned hypoglycemia, genital infections, DKA, and UTI as the most common adverse effects. Some studies also observed renal failure and fractures. Overall, risks were modest and well-tolerated. 

Hypoglycemia: Most common side effect was hypoglycemia. Mild to moderate hypoglycemia was very common. Only some severe hypoglycemia cases were observed, as mentioned in Table 3. A meta-analysis of the sotagliflozin [17] and DEPICT-I [15] trial showed an interesting finding. The overall risk of hypoglycemia was less than that of a placebo, with a 31% lower risk of severe hypoglycemia in sotagliflozin. However, all other studies noticed a slight increase in the episodes of hypoglycemia with SGLT2 inhibitors as compared to placebo.

Infections: SGLT2 inhibitors have not shown a consistent effect on the risk of developing urinary tract infection. In most of the cases, the risk of UTI was higher with placebo. However, there seems to be a relationship between genital infections and SGLT2 inhibitors. In all trials, the incidence was greater with SGLT2 inhibitors. 

Diabetic ketoacidosis: The incidence of DKA with SGLT2 inhibitors is uncommon and has a slightly different clinical presentation than the usual DKA. Most cases of DKA are either due to insulin pump failure or missed insulin dose, but some were due to acute infection. If these identified risk factors are dealt within time, the risk of DKA can be further optimized. Studies have shown that approximately 32% of patients have vomiting problems and that around 35% of people also have glucose levels below 200 mg/dl, although one of the diagnostic criteria for DKA is glucose levels above 250 mg/dl [26]. Hence, blood sugar levels below 250 in patients with DKA can delay the diagnosis and the proper treatment of the patients. 

Multiple studies of SGLT2 inhibitors linked to DKA have been performed. A review article concluded outcomes of eight RCTs which showed that the mean proportion of SGLT2 inhibitor-associated DKA in the placebo group was 0.59% compared to 3.48% and 3.64% respectively in low-dose and high-dose SGLT2 inhibitors [27]. A meta-analysis carried out in 2017 showed that the incidence of SGLT2 inhibitor-associated DKA was less than 1/1000 in randomized controlled trials and 1.6/1000 person-years in cohort studies [26]. Three out of 10 studies in our data reported on euglycemic or hyperglycemic DKA [10, 14, 17]. Most cases had hyperglycemia, but up to 40% had euglycemia. Patients improved with proper treatment in all studies and no complications were observed. No mortality was reported in any of the studies. Findings are explained in Table 4.

**Table 4:** Summary of DKA as seen in clinical trials: incidence, probable cause, and percentage of euglycemic and hyperglycemic cases DKA: diabetic ketoacidosis

Study/ reference	Subjects (N)	DKA (N)	Cause	Percentage of euglycemic and hyperglycemic DKA
Parkins et al., 2014 [10]	40	2	Severe gastroenteritis and insulin pump failure	Hyperglycemic: 50% Euglycemic: 50%
Henry et al., 2015 [14]	35	18	Flu, pneumonia, insulin pump failure or malfunction, inappropriate insulin use	Hyperglycemic: 60% Euglycemic: 40%
Dandona et al., 2018 DEPICT-I [15]	833	27	Insulin dose and insulin pump failure	
Kaku et al., 2019 [16]	40	4	Blood ketone body increases were mild and did not include ketoacidosis	
Musso et al., 2019 [17]	3238	67		Hyperglycemic: 70% Euglycemic: 30%
Mathieu et al., 2020, DEPICT-II [18]	813	21	Insulin pump failure or missed insulin dose	

Effect of SGLT2 Inhibitors on Arterial Efficacy

Diabetics are more prone to major adverse cardiovascular effects (MACE). Therefore, a study was done to assess the effect of SGLT2 inhibitor empagliflozin on endothelial dysfunction and arterial stiffness, which are good predictors of MACE.

Endothelial function assessment was done by brachial artery flow‑mediated dilation (FMD) and reactive hyperemia index (RHI). On the other hand, arterial stiffness was assessed by carotid pulse wave velocity (cPWV), β‑stiffness assessment, and carotid‑femoral pulse wave velocity (cfPWV). The trial was done on 40 patients for 12 weeks. The results showed a marked improvement in all assessments [19].

For endothelial dysfunction, FMD showed improvement up to 2.2-fold and RHI improved up to 1.3-fold. On the other hand, arterial stiffness also showed marked improvement, cPWV, β‑stiffness assessment improved 14.3%, and cfPWV 3.5-fold as compared to placebo respectively. Endothelial function and arterial stiffness improvement are of substantial importance because they can lead to an improvement in cardiovascular risk.

SGLT2 Inhibitors and Renal Function

Raised intraglomerular pressure results in hyperfiltration. It is a common complication of diabetes and can eventually lead to diabetic nephropathy. To see the effects of SGLT2 inhibitors on hyperfiltration, multiple studies have been conducted on drugs like empagliflozin [20] and sotagliflozin [17]. 

A clinical trial conducted to see the effect of empagliflozin on kidney function was done on 40 subjects for eight weeks. It has shown a decrease in GFR (glomerular filtration rate) by 33 mL/min/1.73m^2^ during clamped euglycemia. Similarly, a significant decrease in GFR was observed during clamped hyperglycemia. A modified glucose clamp technique was used in this trial to maintain euglycemic conditions for six hours, followed by hyperglycemic conditions for six hours the next day. This study concluded that short-term treatment with empagliflozin reduces hyperfiltration in subjects with type 1 diabetes. 

On the other hand, a meta-analysis of sotagliflozin [17] has indicated an alternative impact on GFR. It was seen that investigations of under 24 weeks' period indicated improvement in GFR. Unfortunately after 28 weeks, an increment in GFR was seen. Therefore, there is a need for further investigation on sotagliflozin so we can comprehend its effect on GFR. 

Microalbuminuria is an early marker of diabetic nephropathy. A meta-analysis of sotagliflozin has shown a decrease in albumin to creatinine ratio. Decreased albumin levels in diabetic patients give us further proof of the protection that SGTL2 inhibitors provide to kidneys.

Hence, it can be said that SGLT2 inhibitors can have protective effects on kidneys: they decrease microalbuminuria and GFR, but the effects on GFR have to be further explored. 

Fanconi Syndrome 

Fanconi syndrome is a type of renal tubular acidosis, which can be caused by medications. Only one such case was reported after SGLT2 inhibitor use [24]. The patient improved substantially after discontinuation of canagliflozin and after he was hydrated with IV fluids and given subcutaneous insulin and IV potassium phosphate. 

Interpreting evidence and future recommendation

It would be fair to conclude that SGLT2 inhibitors have far more benefits than risks. Their efficacy in controlling HBA1c, significant weight reduction, and their effect on improving arterial function cannot be underestimated. Their role in controlling hyperfiltration and significantly minimizing risks of several life-threatening diseases is also of marked value. These drugs not only help reduce the weight gain secondary to insulin but also tend to reduce morbidity and mortality in the long run. The only significant risk factors are hypoglycemia and DKA. Most cases of hypoglycemia are mild and strict glucose monitoring by the patient can help to solve this problem. For diabetic ketoacidosis, proper doctor and patient education can reduce the risks substantially. The patients should know that in case of fatigue, vomiting, fever, or any presenting symptoms of DKA, their ketone level needs to be tested. However, this does lead us to question whether we have enough knowledge of how DKA caused by SGLT2 inhibitors presents. At which doses is it more likely to occur, which people are susceptible to it, and at which blood sugar level we should suspect DKA? A review needs to be done that can answer these vital questions so that this life-threatening condition can be diagnosed and managed effectively, and so that SGLT2 inhibitors, a groundbreaking discovery, can be prescribed widely, especially in patients with obesity and uncontrolled diabetes.

Strengths and limitations

This review provides a comprehensive overview of not only the safety and efficacy of SGLT2 inhibitors by including randomized control studies but also observational studies showing the benefits and risks of SGT2 inhibitors in type 1 diabetes. It highlights the role of SGLT2 inhibitors in type 1 diabetes. However, there are some limitations to the existing literature review: a collection of studies reported only in English, data obtained from only one database (PubMed), and randomized control trials on sotagliflozin were not included. The only research used is the meta-analysis of the effects of sotagliflozin.

## Conclusions

Our analysis of the published literature concluded that SGLT2 inhibitors' use in type 1 diabetics is associated with more benefits than risks. The typical treatment of type 1 diabetes involves insulin therapy, however, tight glycemic control is very important to prevent microvascular and macrovascular complications. Treatment with insulin could potentially lead to unavoidable hypoglycemic episodes that can prove fatal. Since SGLT2 inhibitors' use is beneficial for type 2 diabetics, its use in type 1 diabetes is also a topic of great interest. Our study has shown that good glucose control, reduction in HBA1c, and weight and blood pressure reduction can not only reduce the anxiety of reaching treatment milestones in type 1 diabetics, but can also prevent major cardiovascular events. Other benefits such as increasing arterial efficacy and improving kidney hyperfiltration have the amazing prospect of improving the overall prognosis of chronically-ill diabetes patients. At the same time, the adverse effects of the drug cannot be overlooked, e.g., UTI, genital infections, hypoglycemia, and DKA. These side effects are modest except for DKA, which may prove to be fatal. But this can be prevented by counseling the patient, educational training to physicians about euglycemic DKA, regular check-ups, and recognition of any symptoms that point towards DKA. With proper care, these medications can be used widely and effectively for patients with DKA. However, deeper and more detailed studies need to be carried out to understand the use of these drugs further in type 1 diabetes patients, with concentrated efforts toward collecting information about SGLT2 inhibitors in DKA.
